# Are Modular Activations Altered in Lower Limb Muscles of Persons with Multiple Sclerosis during Walking? Evidence from Muscle Synergies and Biomechanical Analysis

**DOI:** 10.3389/fnhum.2016.00620

**Published:** 2016-12-09

**Authors:** Tiziana Lencioni, Johanna Jonsdottir, Davide Cattaneo, Alessandro Crippa, Elisa Gervasoni, Marco Rovaris, Emilio Bizzi, Maurizio Ferrarin

**Affiliations:** ^1^Biomedical Technology Department, IRCCS Fondazione Don Carlo Gnocchi OnlusMilan, Italy; ^2^Department of Neurorehabilitation, IRCCS Fondazione Don Carlo Gnocchi Onlus, LaRiCEMilan, Italy; ^3^Department of Multiple Sclerosis, IRCCS Fondazione Don Carlo Gnocchi OnlusMilan, Italy; ^4^Department of Brain and Cognitive Sciences and McGovern Institute for Brain Research, Massachusetts Institute of TechnologyCambridge, MA, USA

**Keywords:** muscle synergy, gait, Multiple Sclerosis, lower limb, EMG

## Abstract

**Background:** Persons with Multiple Sclerosis frequently have gait deficits that lead to diminished activities of daily living. Identification of motoneuron activity patterns may elucidate new insight into impaired locomotor coordination and underlying neural systems. The aim of the present study was to investigate muscle synergies, identified by motor modules and their activation profiles, in persons with Multiple Sclerosis (PwMS) during walking compared to those of healthy subjects (HS), as well as, exploring relationship of muscle synergies with walking ability of PwMS.

**Methods:** Seventeen PwMS walked at their natural speed while 12 HS walked at slower than their natural speeds in order to provide normative gait values at matched speeds (spatio-temporal, kinematic, and kinetic parameters and electromyography signals). Non-negative matrix factorization was used to identify muscle synergies from eight muscles. Pearson's correlation coefficient was used to evaluate the similarity of motor modules between PwMS and HS. To assess differences in module activations, each module's activation timing was integrated over 100% of gait cycle and the activation percentage was computed in six phases.

**Results:** Fifty-nine% of PwMS and 58% of HS had 4 modules while the remaining of both populations had 3 modules. Module 2 (related to soleus, medial, and lateral gastrocnemius primarily involved in mid and terminal stance) and Module 3 (related to tibialis anterior and rectus femoris primarily involved in early stance, and early and late swing) were comparable across all subjects regardless of synergies number. PwMS had shorter stride length, longer double support phase and push off deficit with respect to HS (*p* < 0.05). The alterations of activation timing profiles of specific modules in PwMS were associated with their walking deficits (e.g., the reduction of Module 2 activation percentage index in terminal stance, PwMS 35.55 ± 13.23 vs. HS 50.51 ± 9.13% *p* < 0.05, and the push off deficit, PwMS 0.181 ± 0.136 vs. HS 0.291 ± 0.062 w/kg *p* < 0.05).

**Conclusion:** During gait PwMS have synergies numbers similar to healthy persons. Their neurological deficit alters modular control through modifications of the timing activation profiles rather than module composition. These changes were associated with their main walking impairment, muscle weakness, and prolonged double support.

## Introduction

Persons with Multiple Sclerosis (PwMS) have neuromotor deficits that commonly affect the lower limbs resulting in gait abnormalities that are characteristic of the disease (Kesselring, 2010). Their gait deficit is a major contributor to decreased quality of life, diminished activities of daily living, and loss of employment (Chalah et al., 2015). The mediating efficient communication between the central nervous system (CNS) and neuromotor components, needed to perform the movement, is compromised in persons with Multiple Sclerosis as a consequence of the immune-mediated inflammatory destruction of myelin sheaths, axons, and neurons in cortical and subcortical structures (Haines et al., 2011). Typically in Multiple Sclerosis several lesions occur at different locations within the CNS with a potential impact on the anatomical systems that are involved in walking and in motor control. The resulting impairments can be relative muscle weakness, sensory deficits, spasticity, and fatigue interfering with different aspects of the walking function such as endurance, muscle coordination, and balance with negative impact on activities of daily living (Cattaneo et al., 2014; Frank and Larimore, 2015; Michailidou et al., 2015). Despite the high prevalence of gait disorder in PwMS, there are relatively few reports describing gait parameters and overall these studies have reported slower walking with shorter stride length and prolonged double support phase, independently of their walking speed (Benedetti et al., 1999; Martin et al., 2006; Givon et al., 2009; Cameron and Wagner, 2011; Kasser et al., 2011; Remelius et al., 2012; Lizrova Preiningerova et al., 2015). It has been suggested that these alterations from speed-matched normative values of healthy persons are part of protective strategies used by PwMS to reduce the risk of falling while walking. In fact, a biomechanical characterization of gait patterns of PwMS demonstrated that the same gait impairments, in terms of abnormalities of kinematic, kinetic, and electromyographic (EMG) data, were found in PwMS with different severity of ambulatory deficits (Kelleher et al., 2010). Overall, the gait pattern of PwMS is characterized by a reduced range of hip, knee and ankle motion and by a decreased propulsive force, indicating common pathways in the degeneration of ambulatory ability (Huisinga et al., 2013; Boudarham et al., 2016; Cofré Lizama et al., 2016). Recently, Boudarham and colleagues identified an increased co-activation of agonist-antagonist knee muscles during single support (mid and terminal stance) and in the ankle muscles in PwMS during double support (early stance and pre swing) with respect to healthy subjects that walked at their self selected speeds. The authors hypothesized that this increase in co-activation could be a compensatory mechanism to limit the risk of falling due to altered motor control or that it could also be due to the PwMS walking at a significantly lower speeds than those of healthy subjects (Boudarham et al., 2016). Similar adaptive strategies based on the co-activation of agonist-antagonist muscles have been identified in persons post stroke and attributed to their need to improve ankle stability during crucial phases of the gait, both in weight acceptance and in pre swing (Lamontagne et al., 2000; Chow et al., 2012).

The ability to walk is the result of complex processes involving coordination of multiple systems within the body (e.g., the central nervous system, as well as the, musculoskeletal, cardiovascular, and cardiopulmonary systems). During gait, a person must be able to control a large number of muscles while simultaneously processing sensory information, in order to monitor and refine movements and maintain an upright stance. It has been hypothesized that in neuromuscular control the nervous system relies on muscle synergies (i.e., motor modules), each of which is constituted by two components: The muscle weightings and their temporal activation profiles (Cheung et al., 2009). The motor modules are functional structures related to specific motor patterns, defined as coordinated patterns of muscle activity that flexibly combine to produce functional motor behaviors (Bizzi and Cheung, 2013; Berger and d'Avella, 2014; Ting et al., 2015; d'Avella et al., 2015). The interpretation of those functional structures is, however, still under debate; some consider them as fixed co-excited groups of muscles that contribute toward specific biomechanical function (Ting and Macpherson, 2005; Routson et al., 2014), while others view them as having developed due to optimal control (de Rugy et al., 2013; Ting et al., 2015) or emerging as the result of biomechanical constraints (Kutch and Valero-Cuevas, 2012; Ting et al., 2015). It is likely though that movements, at least for what concerns non-specialized and repetitive actions such as gait or repeated reaching, are governed by modules of muscles functionally organized from the spinal cord (Saltiel et al., 2001; Cheung et al., 2009, 2012; Overduin et al., 2012; de Rugy et al., 2013). Successful walking is thus the product of ongoing modulation of a small set of excitation modules based on task objectives and feedback of the system state (Clark et al., 2010; Dominici et al., 2011; Allen and Neptune, 2012; Gizzi et al., 2012; Chvatal and Ting, 2013; Safavynia and Ting, 2013; Routson et al., 2014; Ting et al., 2015). Through non-negative matrix factorization (NNMF) of EMG signals from 8 leg muscles, three or four muscle synergies have been identified that appear to account for muscle activation during gait in the majority of healthy persons (Clark et al., 2010; Dominici et al., 2011; Fox et al., 2013). Recent studies, focused on muscle synergies during locomotor tasks, have already yielded promising insight into neuromotor control in persons after stroke (Clark et al., 2010; Gizzi et al., 2011; Roh et al., 2013, 2015; Routson et al., 2013, 2014), in children and adults with incomplete spinal cord injuries (Ivanenko et al., 2009; Fox et al., 2013; Hayes et al., 2014), and in children with cerebral palsy (Steele et al., 2015).

Multiple Sclerosis leads to diffuse inflammatory demyelination and neurodegeneration in the brain and spinal cord leading to reduction of white and gray matter integrity relative to healthy subjects. A frequent consequence is the decrease of fiber conduction capacity, which can alter sensory feedback (Rovaris et al., 2000; Lassmann, 2013; Schlaeger et al., 2014). All of the above could lead to alteration in muscle synergies modifying both the muscle weightings and the related activations profiles because they are respectively attributed to a lower level (e.g., spinal cord) and a higher level (e.g., cortical structures) of motor control.

Knowledge of neuromuscular coordination in PwMS detectable with the analysis of muscle synergies, could offer clinicians insight into both underlying neural strategies for movement and functional outcomes of muscle activity in that population (Safavynia and Ting, 2013). Such information could be useful in rehabilitation treatments and could inform diagnostic tools and evidence-based interventions specifically targeted to deficits of the individual PwMS. Further, the investigation of the motoneuron activity patterns that characterize walking in PwMS may elucidate new insight into how changes in motor performance are related to abnormal muscle recruitments and deficits in the underlying neural systems.

While sensory changes, lower extremity weakness and spasticity are thought to contribute to most of the gait deficits observed in Multiple Sclerosis (Cameron and Wagner, 2011) the relationship of such observations to underlying muscle synergies is unknown. The use of motion analysis systems can identify neuromotor mechanisms underlying gait dysfunction and how they relate to muscle activation (Cofré Lizama et al., 2016). Until now, most studies compared gait patterns between PwMS and healthy persons, investigating directly the EMG activation patterns of the main lower limb muscles: Rectus femoris, hamstrings, tibialis anterior, and gastrocnemius (Cofré Lizama et al., 2016). However, a closer view on how muscle groups functionally act together during gait is still missing. Consequently, the impact of the underlying neurological deficit on number, composition and activation of muscle synergies and their relationship to gait parameters is not well understood in PwMS. Modules of muscle synergies have been hypothesized to be specified at lower neural centers (e.g., subcortical and spinal cord) while modules activation profiles are supposedly influenced by signals from CNS higher centers (Cheung et al., 2009; Overduin et al., 2012; Waters-Metenier et al., 2014; Rana et al., 2015). Since PwMS have diffuse damages both at cortical and spinal cord level, it is possible that they show changes in both muscle weightings and activation profiles of muscle synergies.

Based on the above, the aim of this study was to investigate muscle weightings and their activation profiles during locomotion in PwMS and compare them with those of healthy persons, as well as, exploring the relationships between muscle synergies and walking ability in PwMS.

## Materials and methods

### Participants and clinical setup

A convenience sample of 23 persons diagnosed with Multiple Sclerosis was recruited from the inpatient population of Don Carlo Gnocchi Foundation Onlus, “Santa Maria Nascente” Center, Milan, Italy between May 2014 and June 2015. After screening for inclusion/exclusion criteria, participants in this cross-sectional study included 17 adults with Multiple Sclerosis [age (mean ± SD) 51.7 ± 11.0 yrs, 11 females, body weight 66.3 ± 10.1 kg, body height 165.1 ± 7.8 cm] and 12 age-matched healthy adults (47.2 ± 5.0 yrs, 5 females, body weight 67.9 ± 14.8 kg, body height 168.8 ± 9.8 cm). Inclusion criteria for PwMS included having a definite diagnosis of Multiple Sclerosis, an Expanded Disability Status Scale (EDSS) score of 7 or lower, capacity to stand upright for 30 s without assistance, capacity to walk without support for 10 m on a level surface, capacity to understand and follow instructions, and stability of neurological condition. Criteria of exclusion were any other significant non-Multiple Sclerosis related impairment affecting walking. Healthy controls (HS), matched for age, did not have any neuromuscular and balance deficits that could interfere with their gait, and exhibited normal joint range of motion and muscle strength. All subjects participating in the study provided informed consent to the protocol approved by the local Ethical Committee (Comitato Etico Fondazione Don Carlo Gnocchi).

### Evaluation protocol

Kinematic, kinetic, and electromyography data were collected from 17 subjects affected by Multiple Sclerosis, and from 12 healthy controls. Kinematic data were collected using a 9-camera SMART-E motion capture system (BTS, Milano, Italy) sampling at 60 Hz; while a force plate (Kistler, Winterthur, Switzerland), with 960 Hz sampling frequency, provided ground reaction force (GRF). An 8-channel EMG system (BTS, Milano, Italy) was used to record EMG data, at 1000 Hz frequency, from the following muscles: Tibialis anterior (TA), soleus (SO), medial gastrocnemius (MG), lateral gastrocnemius (LG), vastus medialis (VM), rectus femoris (RF), semitendinosus (SE), gluteus medius (GM). The EMG probes were placed on the dominant side (the leg that was used to kick a ball) of control subjects and on the most affected side of PwMS selected according to item 13 of the Berg Balance Scale [BBS, Standing unsupported one foot in front (Berg et al., 1992)]. All participants performed five walking trials at their natural speed and the healthy subjects also performed trials at lower speeds. Since there are speed-dependent effects on the timing patterns of muscle activity and on kinematic and kinetic parameters (Stoquart et al., 2008; Jonsdottir et al., 2009; Clark et al., 2010; Routson et al., 2014), only trials of healthy subjects with speed smaller than 0.75 m/s, correspondent to 90% of the maximum speed value of PwMS, were selected for the kinematic, kinetic, and muscle synergies analysis.

The total-body LAMB marker set was adopted, which includes 29 retro-reflective markers (12 mm diameter) positioned on the head, upper limbs, trunk, pelvis, and lower limbs (Rabuffetti and Crenna, 2004). After data acquisition, the markers' coordinates were low-pass filtered at a cut-off frequency of 6 Hz. Anthropometric parameters of each subject were computed from markers' positions recorded during the calibration trial, and used for the estimation of internal joint centers and inertial parameters of body segments. Inverse dynamics derived from the recorded ground reaction forces and anthropometric measures were used to compute moments and powers at the ankle, knee, and hip joints. Each trial included the single gait cycle performed on the force plate. For each patient, the average value of selected parameters and the average pattern of kinematic/kinetic variables across trials were computed.

The calculated spatio-temporal, kinematic and kinetic parameters, known to be related to gait ability in PwMS, are reported in Table 1 (Kaipust et al., 2012; Huisinga et al., 2013; Cofré Lizama et al., 2016; Kempen et al., 2016).

**Table 1:** **Mean values (SD) of spatio temporal, kinematic, and kinetic parameters of healthy subjects (HS) and persons with Multiple Sclerosis (PwMS) as whole groups**.

**Parameters**	**HS**	**PwMS**
**SPATIO-TEMPORAL**		
Gait speed (m s^−1^)	0.550 (0.064)	0.500 (0.223)
Cadence (steps min^−1^)	72 (7)	73 (19)
Stride length (m)	1.092 (0.094)	0.785 (0.204)^*^
Double support loading response (%)	16 (1)	21 (7)^*^
Double support pre swing (%)	16 (2)	20 (7)^*^
Heel rise at terminal stance (m)	0.088 (0.011)	0.083 (0.017)
Toe clearance in swing (m)	0.127 (0.019)	0.087 (0.033)^*^
**KINEMATICS**		
Peak dorsiflexion in terminal stance (deg)	−6 (3)	−12 (6)^*^
Peak dorsiflexion in swing (deg)	−15 (4)	−26 (7)^*^
Plantar flexion angle at foot contact (deg)	−22 (3)	−31 (7)^*^
Ankle flexion ROM (deg)	33 (7)	26 (6)^*^
Peak knee flexion in swing (deg)	59 (4)	47 (14)^*^
Peak knee extension in terminal stance (deg)	7 (4)	2 (11)
Knee flexion ROM (deg)	56 (5)	46 (15)^*^
Peak hip flexion in swing (deg)	32 (2)	34 (6)
Hip flexion ROM (deg)	38 (5)	35 (11)
**KINETICS**		
Peak ankle dorsiflexor power in terminal stance (w/kg)	−0.514 (0.177)	−0.517 (0.352)
Peak ankle plantarflexor power in pre swing (w/kg)	1.706 (0.481)	1.357 (1.220)
Ankle positive mechanical work (w/kg)	0.291 (0.062)	0.181 (0.136)^*^
Ankle negative mechanical work (w/kg)	−0.134 (0.036)	−0.119 (0.051)
Peak knee flexion power in loading response (w/kg)	−0.192 (0.188)	−0.319 (0.272)
Peak knee flexion power in pre swing (w/kg)	−0.262 (0.183)	−0.238 (0.243)
Peak knee flexion power in swing (w/kg)	−0.214 (0.072)	−0.312 (0.294)
Knee positive mechanical work (w/kg)	0.038 (0.032)	0.079 (0.093)
Knee negative mechanical work (w/kg)	−0.107 (0.029)	−0.120 (0.083)
Peak hip flexor in loadin response (w/kg)	0.282 (0.194)	0.292 (0.207)
Peak hip extensor in terminal stance (w/kg)	−0.248 (0.083)	−0.370 (0.315)
Peak hip flexor in pre swing (w/kg)	0.201 (0.073)	0.308 (0.300)
Hip positive mechanical work (w/kg)	0.090 (0.047)	0.125 (0.073)
Hip negative mechanical work (w/kg)	−0.077 (0.047)	−0.072 (0.055)

* *p < 0.05: Statistically significant difference between PwMS and HS*.

### Data processing and analysis

The EMG signal was high pass filtered with a cutoff frequency of 40 Hz, rectified, and low pass filtered with a cutoff frequency of 10 Hz, using a 4th order Butterworth filter (Routson et al., 2013). In order to not alter the variability in EMG, the signal of each muscle was amplitude-normalized to its peak value across all trials (Clark et al., 2010; Routson et al., 2014). All data were time normalized to 100% of the gait cycle and subsequently averaged. The muscle synergies were extracted using non-negative matrix factorization [NNMF, (Lee and Seung, 1999)] from averaging all recorded gait cycles. Briefly, for each subject, the EMGs were combined into an *m* × *t* matrix, where *m* indicates the number of muscles and *t* is the time base (*t* = averaged stride × 101). The synergy extraction was repeated 50 times. The solution that accounted for >90% of the EMG variability for each muscle was selected thus obtaining two matrices for each extracted muscle synergy: An *m* × *1* array, which specifies the relative weighting of each muscle in the module (module composition) and an *1* × *t* array, which specifies the activation timing profile of the module.

To enable a one-to-one comparison of module composition (related to muscle weightings) for each subject in the Multiple Sclerosis group with that of the HS group, each patient's module composition was compared to the averaged module composition of those in the corresponding comparison HS group (i.e., all PwMS vs. all HS, PwMS with 3 modules vs. HS with 3 modules, PwMS with 4 modules vs. HS with 4 modules). Higher correlations [Pearson's correlation coefficient (*r*), with 1.0 being a perfect association with the control group mean] indicated a higher similarity in module compositions.

To assess the differences in activation timing profiles, for each subject (both in the PwMS and HS groups), each synergy's activation profile was integrated over 100% of the gait cycle (activation area) and then the percentage of such activation area (reported in the text and in the bar graphs as activation percentage index) was calculated within each of the following six phases of the gait cycle (Routson et al., 2014): Early Stance (P1), Mid Stance (P2), Terminal Stance (P3), Pre Swing (P4), Early Swing (P5), and Late Swing (P6).

Biomechanical and EMG measures were analyzed using Matlab® (MathWorks Inc., MA, USA).

### Statistical analysis

All statistical analyses were performed using Matlab® for Windows (MathWorks Inc., MA, USA).

As Module 2 and 3 were found to be similar across all subjects, independent of the number of synergies (see Results section), all PwMS were compared to all healthy subjects using the unpaired *t* test for the parameters related to the muscle synergies (regarding module composition and timing of Modules 2 and 3) and to kinematics and kinetics. Conversely, for the comparison of Modules 1 and 4, the data of PwMS were compared with the HS subgroup having the same number of synergies.

The values of analyzed parameters were summarized and tabulated as means and standard deviation (SD). *P*-values < 0.05 were considered statistically significant and, due to the many tested individual hypotheses in the comparison between HS and PwMS, Holm-Bonferroni correction was applied.

## Results

**Figure 1:**
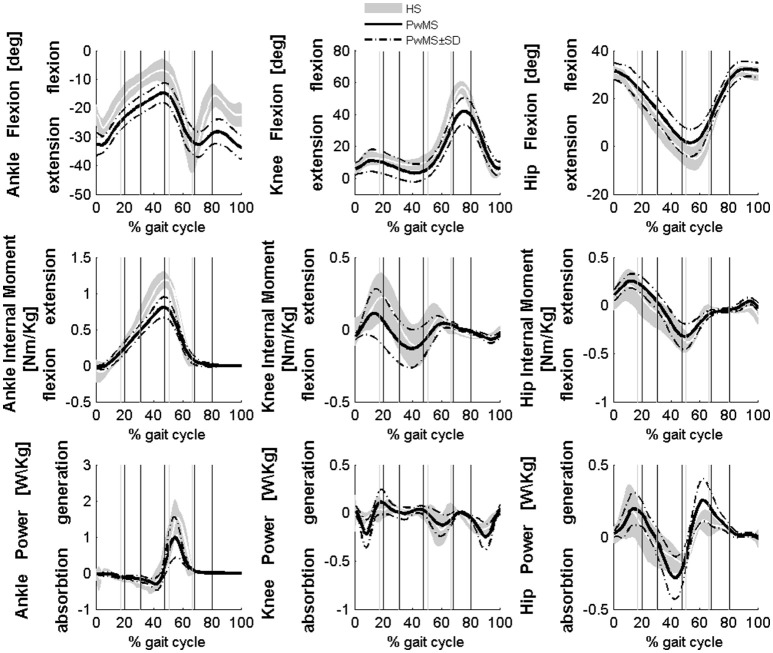
**Averaged kinematics and kinetics of PwMS with three and four muscle synergies during walking are reported in a solid black line**. Dashed black lines represents ± SD of PwMS curves. Vertical lines (solid gray line—healthy subjects and solid black line—PwMS) indicate the phases of normalized gait cycle, Early Stance, Mid Stance, Terminal Stance, Pre Swing, Early Swing, Late Swing. Range of normality is reported in gray.

All seventeen PwMS and 12 HS completed the whole testing protocol. The mean onset of disease in PwMS was 16.7 ± 7.2 (mean ± SD) years and the average EDSS was 5.8 ± 0.8. EDSS, BBS total score, and gait velocity were comparable in PwMS with three and four modules (respectively, EDSS 5.7 ± 1.0 and 5.8 ± 0.9, *p* > 0.05; BBS total score 46.1 ± 4.6 and 42.7 ± 10.7, *p* > 0.05; gait velocity 0.53 ± 0.26 and 0.45 ± 0.18 ms^−1^, *p* > 0.05), therefore spatio-temporal, kinematic, and kinetic parameters were averaged among all PwMS. The averaged number of trials for HS included in the analysis was 4.1 ± 1.4. Comparing groups at matched speed, double support time was significantly longer in PwMS than in HS, stride length was shorter and toe clearance in swing was lower. Range of motion in ankle and knee was smaller in PwMS (see Figure 1 and Table 1) while no significant differences emerged at level of the hip joint, although PwMS tended to walk with less hip extension at toe-off (end of pre swing) and more hip flexion during swing as already evidenced (Benedetti et al., 1999). The knee and ankle flexion in swing were significantly reduced in PwMS, and peak dorsiflexion in terminal stance and plantarflexion angle was significantly smaller at foot contact. There were no significant differences in power absorption and generation at hip and knee level while positive work produced at the ankle was significantly reduced in PwMS compared to HS indicating an important propulsive force deficit (Figure 1 and Table 1).

### Number of muscle synergies

The total variance in the reconstructed EMG data from the extracted muscle synergies accounted for was comparable between HS and PwMS (*p* > 0.05, 92.4 ± 1.5 vs. 93.3 ± 2.0%, respectively). The number of muscle synergies identified was similar in PwMS and HS, 58% of HS had 4 modules and 42% had 3 modules while 59% of PwMS had 4 modules and 41% had 3 modules. In both PwMS and HS with four muscle synergies, Module 1 consisted mainly of proximal muscle activity from VM (knee extensor), RF and GM (hip extensor and abductor), primarily involved in early stance and late swing (P1 and P6) to prepare the leg for weight acceptance (Winter and Yack, 1987); Module 2 consisted mainly of muscle activity from SO (ankle plantarflexor), MG and LG (ankle plantarflexors and knee flexors) primarily involved in the forward propulsive phase (P2 and P3); Module 3 consisted mainly of muscle activity from TA (ankle dorsiflexor), and RF (knee extensor and hip flexor) activation primarily involved in early stance and swing (P1, P5, and P6) related to control during ground clearance of the leg; Module 4 consisted mainly of hamstring activity from SE (hip extensor and knee flexor), primarily involved in the early stance (P1) and late swing (P6), respectively to extend the hip and decelerate leg swing. Compared to other works in the literature (Clark et al., 2010; Dominici et al., 2011; Gizzi et al., 2011) the subjects in the present study showed a participation, although minor, of the GM in Module 3. However, this is in agreement with other works that show that activity of the gluteus begins at terminal swing, in preparation for the heel strike and continues throughout the first half of the stance phase (Benedetti et al., 2012).

### Muscle synergies 2 and 3

**Figure 2:**
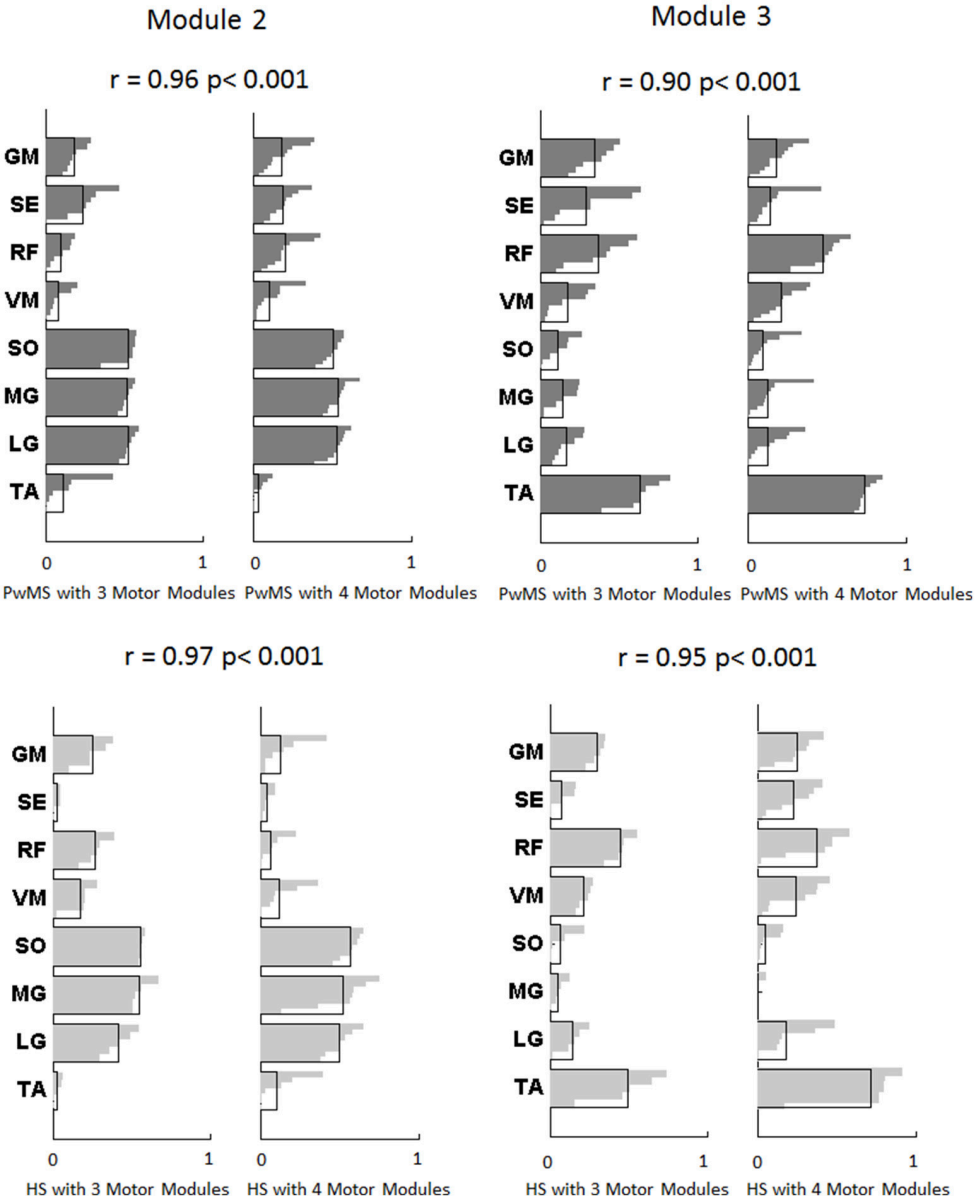
**Modules composition (muscle weightings) of muscle synergies of persons with Multiple Sclerosis are depicted in darker gray (above) and for healthy subjects in lighter gray (below) related to Module 2 for forward propulsion and Module 3 for control of ground clearance of the leg**. The values of Pearson's correlation (*r*) and the correspondent *p*-values (*p*) between PwMS with three synergies and PwMS with four synergies, and between HS with three synergies and HS with four synergies are reported. The activation profiles of Module 2 and 3 are reported in Figure 3. TA, tibialis anterior; SO, soleus; MG, medial gastrocnemius; LG, lateral gastrocnemius; VM, vastus medialis; RF, rectus femoris; SE, semitendinosus; GM, gluteus medius.

**Figure 3:**
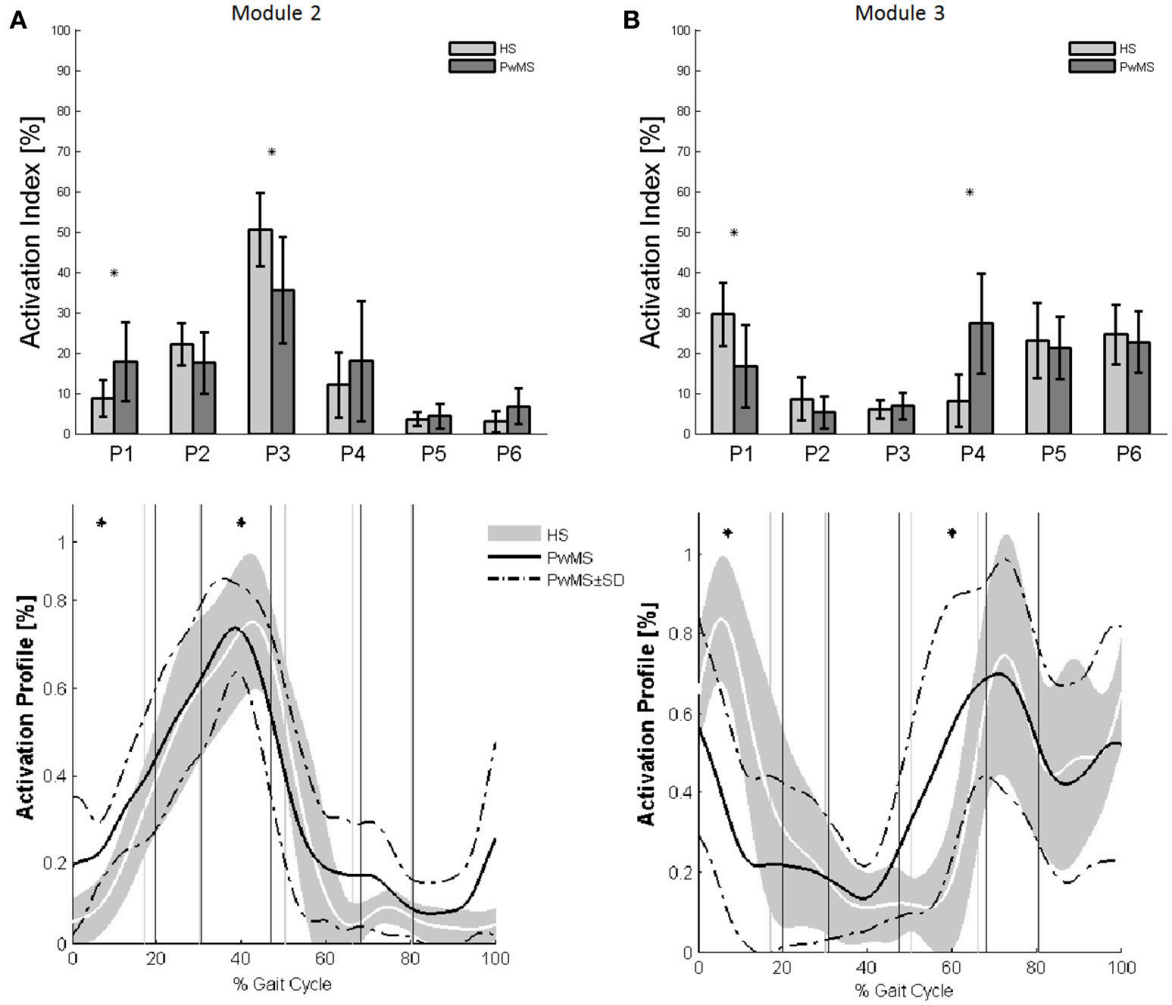
**The upper panels show the bar graphs of the activation percentage indexes during gait phases of (A)** Module 2 for forward propulsion and of **(B)** Module 3 for the control of the ground clearance of the leg in healthy subjects (HS in lighter gray) and in persons with Multiple Sclerosis (PwMS in darker gray) as whole groups. The lower panels show the averaged activation timing profiles of **(A)** Module 2 and **(B)** Module 3 in HS and in PwMS. The solid black line represents the averaged profile of PwMS and the dashed black lines represent ± SD of PwMS curves. Vertical lines (solid gray line—healthy subjects and solid black line—PwMS) indicate the phases of normalized gait cycle. Range of HS normality is reported in gray. P1, Early Stance; P2, Mid Stance; P3, Terminal Stance; P4, Pre Swing; P5, Early Swing; P6 Late Swing. ^*^p < 0.05: Statistically significant difference between PwMS and HS.

Muscle weightings of Module 2 and Module 3 were found to be comparable across all subjects regardless of synergies number, both in PwMS and HS (Figure 2). Therefore, the analysis of Modules 2 and 3 was done considering all PwMS compared to all HS. The similarity of Module 2 and Module 3 of PwMS with the average module composition of HS was high (>0.70+, *p* > 0.05). Through analysis of activation timing profiles, important statistically significant differences, expressed by the activation percentage indexes, were found between PwMS and HS in Module 2 during the stance phases of early and terminal stance (P1 and P3, Figure 3A). PwMS showed a statistically significant larger activation in P1 than controls, consisting of larger activation of SO, MG and LG that normally contribute little in this phase. On the contrary in terminal stance (P3), where SO, MG, and LG should be active, these muscles were instead significantly less active in PwMS than in HS (Figure 3A). Considering Module 3, PwMS had a statistically significant smaller activation in loading response (P1) and greater activation in pre swing (P4), during late push off, than HS (Figure 3B). Regarding the intersubject variability, the average value for SD of the activation profiles of Modules 2 and 3 were similar between PwMS and HS (Figure 3, normalized units, Module 2: 0.242 PwMS vs. 0.248 HS; Module 3: 0.161 PwMS vs. 0.169 HS), indicating that PwMS did not show greater variability compared to the HS.

### Muscle synergies 1 and 4

**Figure 4:**
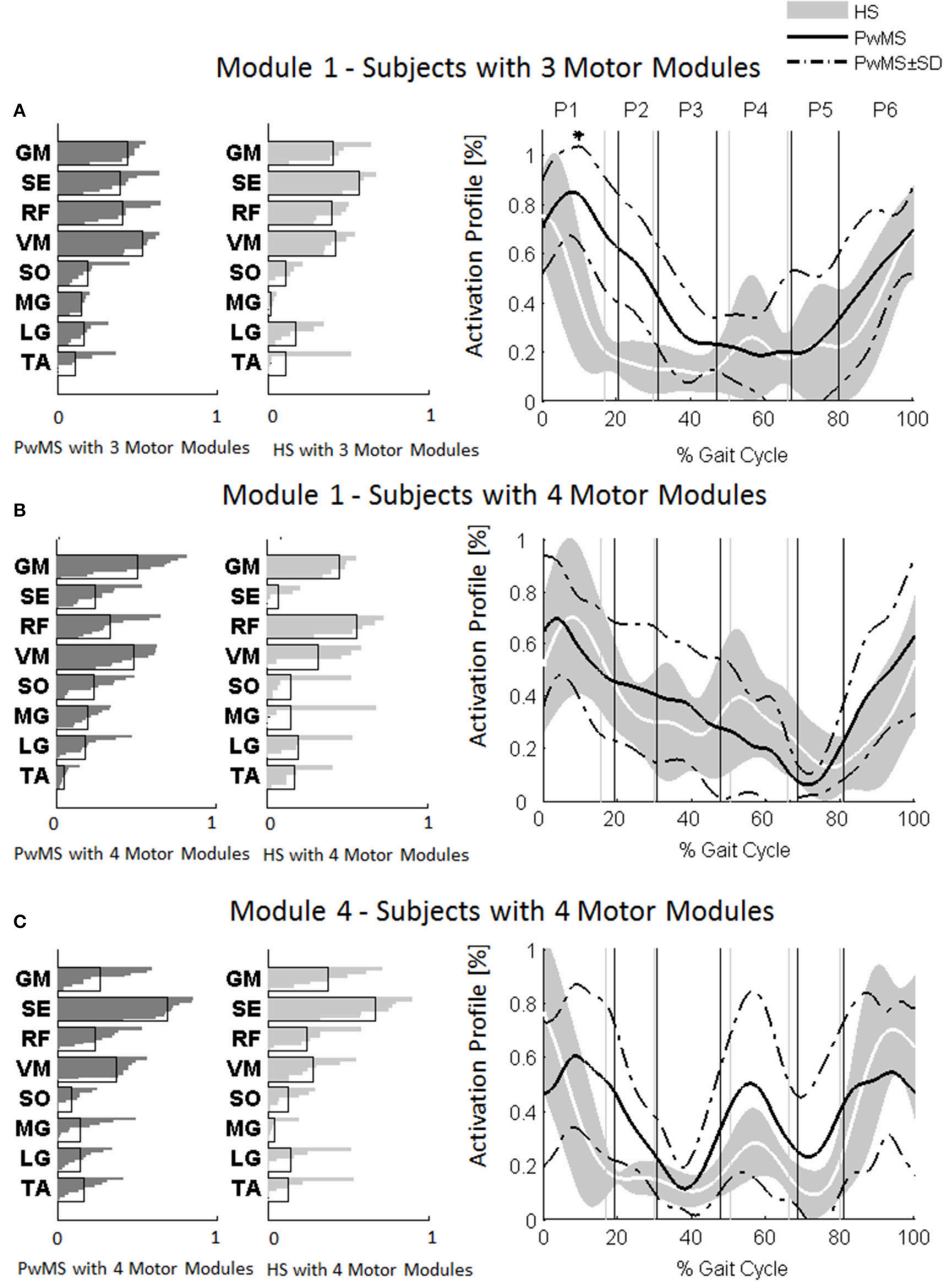
**Muscle weightings are depicted on the left in darker gray for persons with Multiple Sclerosis (PwMS) and in lighter gray for healthy subjects (HS)**. The activation timing profiles are reported on the right: **(A)** Module 1 in HS and PwMS with three muscle synergies; **(B)** Module 1 and **(C)** Module 4 in HS and PwMS with four muscle synergies. The solid black line represents the averaged profile of PwMS and the dashed black lines represent ± SD of PwMS curves. Vertical lines (solid gray line—healthy subjects and solid black line—PwMS) indicate the phases of a normalized gait cycle. Range of HS normality is reported in gray. ^*^p < 0.05: Statistically significant difference between PwMS and HS. TA, tibialis anterior; SO, soleus; MG, medial gastrocnemius; LG, lateral gastrocnemius; VM, vastus medialis; RF, rectus femoris; SE, semitendinosus; GM, gluteus medius. P1, Early Stance; P2, Mid Stance; P3, Terminal Stance; P4, Pre Swing; P5, Early Swing; P6, Late Swing.

Investigation of module composition across persons with three and four muscle synergies revealed that when there were only three muscle synergies, Module 1 appeared to be a merging of Modules 1 and 4 (Figure 4).

**Figure 5:**
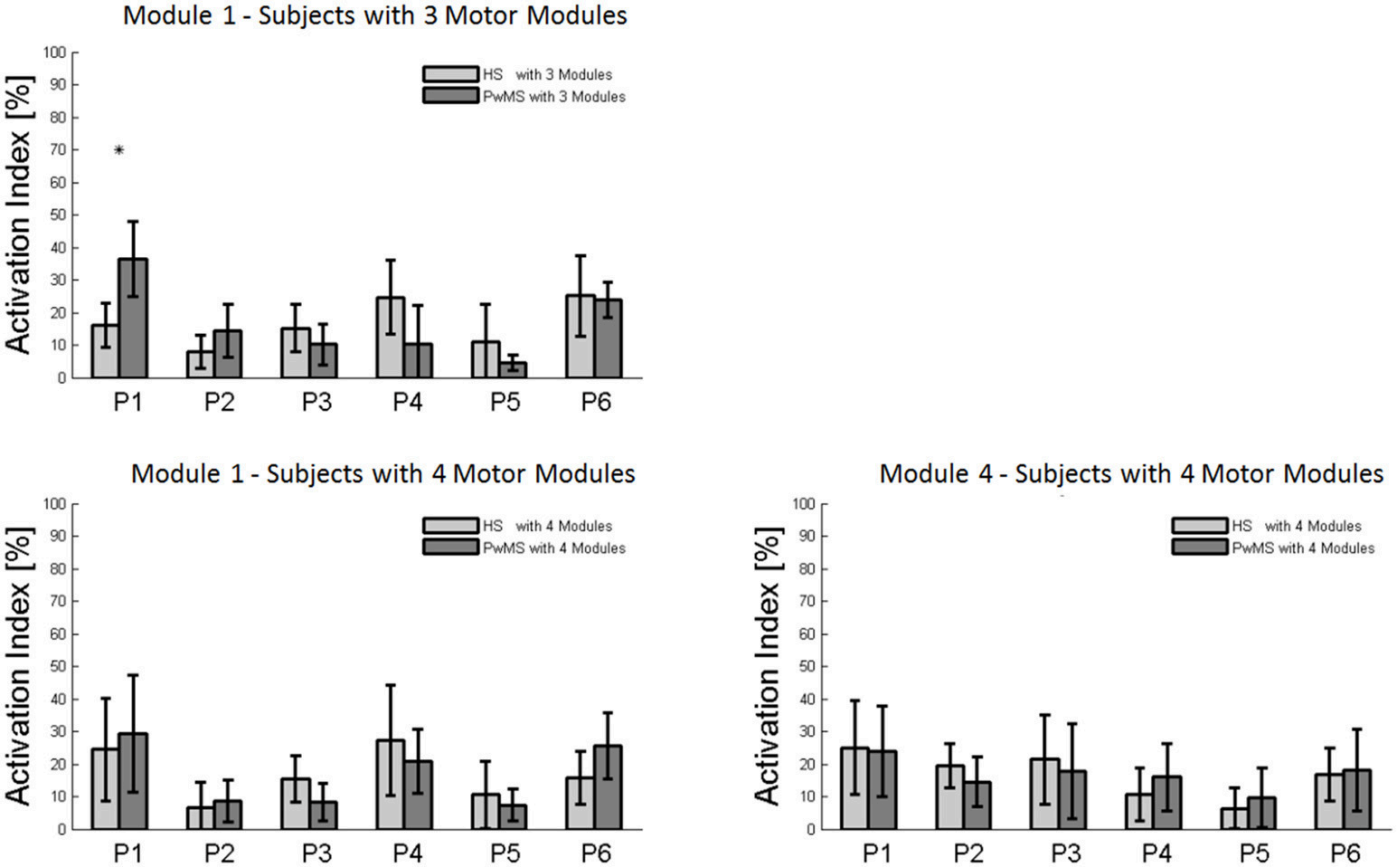
**Bar graphs of the activation percentage indexes during gait phases of Module 1 in healthy subjects (HS) and persons with Multiple Sclerosis (PwMS) with three muscle synergies and of Module 1 and Module 4 in healthy subjects (HS) and persons with Multiple Sclerosis (PwMS) with four muscle synergies**. ^*^p < 0.05: Statistically significant difference between PwMS and HS.

The composition of Module 1 in PwMS with three muscle synergies had high similarity with the average module composition of the HS group with the same number of synergies (0.77 ± 0.10, *p* > 0.05 Figure 4A left). Also PwMS with four muscle synergies showed similar composition of Module 1 and Module 4 (>0.70+ *p* > 0.05, Figures 4B,C left) to healthy controls with four muscle synergies. Comparing the activation percentage indexes of Module 1 of PwMS and HS with three synergies, the only significant difference was found in an excessive activation of this proximal synergy in PwMS during loading response (Figure 5). No statistically significant differences were found comparing the activation percentage indexes of Modules 1 and 4 of PwMS and HS with four synergies (Figure 5). Similar to intersubject variability findings for muscle synergies 2 and 3, the variability of the activation profiles of Module 1 and Module 4 among PwMS were comparable to that among HS (average value for SD in normalized units, Module 1: PwMS with three and four synergies 0.205 0.138 vs. HS with three and four synergies 0.199 0.144; Module 4: 0.117 PwMS vs. 0.123 HS).

## Discussion

In this study we investigated muscle synergies and locomotor ability in persons moderately affected by Multiple Sclerosis and in age-matched healthy subjects at similar gait speeds. The relationship between neuromotor impairments, expressed by muscle synergies, and locomotor ability was further explored in the group of PwMS. Gait analysis confirmed the typical locomotor pattern of persons with Multiple Sclerosis, in fact our sample of PwMS overall adopted the strategy of significantly prolonged double support periods (early stance and pre swing) and shorter strides compared to healthy subjects. PwMS also showed reduced knee flexion and ankle dorsiflexion during swing that led to decreased toe clearance, confirming that PwMS are subjects exposed to a high risk of falling, as well as an important reduction of propulsive work at the ankle. These findings are consistent with co-contraction and muscle weakness as already suggested in the literature (Benedetti et al., 1999; Kasser et al., 2011).

### Muscle synergies and gait ability in healthy subjects and PwMS

Similarly to what has been previously found in post stroke subjects (Clark et al., 2010), our findings suggest that, in persons moderately affected by Multiple Sclerosis and with a slow gait velocity, motor module composition (muscle weightings) remains consistent with that of healthy subjects walking at a similar gait velocity, regardless of the number of muscle synergies, even when biomechanical factors are compromised. The absence of effect of Multiple Sclerosis on number and composition of motor modules, supports the hypothesis that muscle weightings are modulated as distinctive functions of locomotion speed, as suggested by Gonzalez-Vargas et al. (2015).

A diffused neurological damage such as that of PwMS appears to not necessarily result in fewer muscle synergies. The frequencies of persons having four muscle synergies and three muscle synergies in our sample of healthy subjects (respectively 58 and 42%) and PwMS (59 and 41%), was similar to that found in the 20 healthy subjects recruited by Clark et al. (2010) (55 and 38%). The fact that the percentage of PwMS that had three muscle synergies is similar to that of healthy subjects and that there were no differences in disability, as expressed by the EDSS scores between PwMS groups that had three and four muscle synergies, indicates that in general the number of muscle synergies is not altered in PwMS regardless of neuromotor deficits. This is in contrast with findings from post-stroke subjects that indicated a correlation between locomotor pattern and underlying muscle synergies number, given that post-stroke persons with worse performance showed fewer modules (Clark et al., 2010; Allen et al., 2013). This discrepancy can be related to the different impacts of the distinctive neurological damages underlying these two disorders. The merging or fractionation of muscle synergies in persons after stroke (Clark et al., 2010; Allen et al., 2013) suggests a unique effect of stroke on synergies, while the diffuse central and spinal neuronal damage and atrophy characteristic of Multiple Sclerosis may not impact directly on the number of synergies (related to synergy complexity), indicating a preserved independence of neural control signals (Schlaeger et al., 2014).

As already observed in post stroke subjects (Clark et al., 2010), the scrutiny of the pattern of muscle weightings and activation profiles of PwMS with three muscle synergies indicates an apparently functional merging of Modules 1 and 4 of subjects with four synergies (Figures 4B,C) with the semitendinosus muscle acting simultaneously to the quadriceps muscles (RF and VM) during early stance and swing phases. These characteristics appear not to impact on locomotor performance as evidenced by similar EDSS scores between PwMS with three and four synergies. What instead did distinguish PwMS from healthy controls were different alterations of muscle synergies activation timing profiles during locomotion resulting in kinematic and kinetic profiles characteristic of PwMS, as discussed in the following paragraph.

### Relationship between motor synergies and biomechanical variables in PwMS

An important result of this study is the relationship between the measured characteristics of the gait pattern of PwMS and their neurological impairment. This became apparent in the analysis of the muscle synergies' activation percentage indexes over the gait cycle. PwMS showed an inappropriate timing and non-functional profile of activation of muscle synergies that may be related to the typical prolonged double support time strategy. In particular, differences were found in muscle synergy 2 and 3 that are associated with distal muscle activation as hereafter discussed.

During terminal stance (P3) PwMS showed a significant reduction in activation of Module 2 because this synergy is activated in reduced time during the single support phase [activation percentage indexes of Module 2 (Figure 3A)]. This deficit leads to a propulsion force reduction in PwMS with respect to HS (Table 1 Ankle Positive Work parameter and Figure 1) in agreement with recent work of Honeine and colleagues that highlights the importance of triceps surae in kinematics and kinetics control of gait (Honeine et al., 2014). In fact in healthy subjects, during terminal stance, the action of plantarflexors increases to restrain the continued forward momentum of the body (Inman et al., 1981; Winter, 1987; Honeine et al., 2014). The alteration of the control of this mechanism results in a non-functional ankle position at the beginning of the propulsive phase, as expressed by the significant reduction of the peak dorsiflexion in terminal stance (Table 1). With regard to Module 3, that represents the ground clearance of the leg, the activation timing indexes were statistically different from those of HS during double support, early stance and pre swing phases. The PwMS were not able to control distally the impact with the ground during loading response (Figure 3B) and had compromised ankle positioning at the beginning of the stride (Figure 1), thus altering the subsequent phases. In fact PwMS showed reduced clearance in swing (Table 1) that consequently lead to poor control of the ankle joint at the beginning of the stride. The lack of proper activation of Module 3 required, as a compensation, greater activation of the antagonist Module 2 that was more active during loading response in PwMS than in HS (P1 Module 2 and 3, Figure 3). The relationship of agonist-antagonist synergies was repeated in pre swing phase but with a different ratio. During pre swing phase the activation of the knee extensor should be limited to allow the knee to flex (Gage, 1990; Rodgers, 1995); instead PwMS seemed to anticipate significantly the activation of Module 3 with an abnormal force during this phase (Figure 3B) possibly leading to a stiff knee during gait (Figure 1). This behavior is consistent with a protective strategy typical of poor balance as already suggested in PwMS (Benedetti et al., 1999; Boudarham et al., 2016) that would be adopted because of weak ankle muscles, important for stability of the knee in the stance and pre swing phase. The finding of increased co-activation of agonist-antagonist synergies (Module 2 and 3) in PwMS compared to those of HS walking at similar speeds suggests that this mechanism may be part of a characteristic pattern implemented as a result of the neurological damage and environmental requirements.

Muscle synergies related to proximal muscle activation (i.e., Modules 1 and 4) in PwMS were not statistically different over the gait cycle from those of HS (Figure 5), although there were apparent differences that may explain differences in gait ability. Module 1, related to weight acceptance, did not reach a clear peak of maximum activity during early stance (P1) in PwMS as happened in HS (Figure 4C). The deficit in extensor muscular activity during P1 (reduced peak knee flexion power in the loading response, Figure 1 and Table 1) in PwMS may indicate a lack of control of this phase due to ankle instability (deficit in control of Module 3, see discussion above) and weakness of knee muscles (weakness of quadriceps present in Module 1) characteristic of persons with MS (Wens et al., 2014). As compensatory strategy, Module 4, related to leg deceleration and antagonist of Module 1, rightly active in the last part of swing phase, continued to be active in an excessive manner during early and mid stance (Figure 4B) probably to contain hip extension and to stabilize the knee. These motor behaviors were associated with an increase of the phase of double support in loading response (P1). These findings confirm that persons with Multiple Sclerosis tend to compensate for muscle weakness by keeping muscles active for longer time and is consistent with suggestions of Martino and colleagues that the nervous system copes with unstable conditions by prolonging the duration of basic muscle activity patterns (Martino et al., 2015). The above mentioned behaviors did not emerge statistically from the analysis of the activation percentage indexes, probably due to the low number of subjects in the individual groups (group with four synergies, Figure 5).

As discussed above, in subjects with three muscle synergies, Module 1 and 4 appear to have lost their independence and seem to be merged into a single module, Module 1, as already suggested in post stroke subjects (Clark et al., 2010). There is an apparent functional reorganization in the most proximal part of the lower limb that happens in both PwMS and HS without influencing their locomotor ability, with thigh muscles (SE, VM, and RF) acting simultaneously and not independently. In PwMS with three synergies, the need to further activate the proximal part to compensate for the distal deficit was evident. In fact there was an excessive activity of Module 4 in weight acceptance which was maintained in mid stance in an abnormal manner with respect to HS with the same number of muscle synergies. The activation percentage index of Module 1 in the group with three synergies (Figure 5) was higher with respect to HS with three synergies during weight acceptance, while this was not evident in the group with four muscle synergies. It may be that, having a simpler control scheme, the group with three muscle synergies compensated for balance instability during gait by increasing activity of proximal muscles.

As discussed in the introduction section, the literature supported by several decades of evidences has led to the conclusion that automated walking is largely accomplished by a network of spinal neurons [central pattern generator (CPG)] which generate the rhythm for locomotion under the influence of the CNS. The exact roles and interactions between all brain structures and CPG neurons are not yet fully known, however it is generally accepted that several areas of the brain are capable of inducing and/or changing CPG-driven activity (Cheron et al., 2012; Guertin, 2012). In fact, there is evidence of an influence of locomotor activity level and timing by neocortical and corticospinal tracts (Bretzner and Drew, 2005; Tassinari et al., 2009). The PwMS involved in the present study showed an apparently intact modular organization that was appropriate for a given speed, while there were differences from HS in activation timing profiles of muscle synergies, indicating that PwMS may have a problem in the generation of rhythm of locomotion. Other authors studying restless leg syndrome have similarly suggested an alteration of CPG signals in PwMS (Chervin et al., 2003; Tassinari et al., 2005, 2009; Guertin, 2012). A precise cortical motor control of timely muscle activation during steady gait would need accurate information from peripheral sensory feedback, it would need a normal nerve conduction velocity, intact cortical regions normally involved in locomotor control (mesencephalic locomotor region and subthalamic region) and an intact cerebellum involved in control and coordination of balance and locomotion (Clark, 2015). It is likely that, in the present study population of PwMS, with moderate functional deficits, several of the above mentioned structures involved in motor control were impaired causing a faulty interaction between brain, spinal cord pattern generator, interneurons and motorneurons and sensory feedback during gait. This would result in less precise motor control as reflected in changes in activation profiles of muscle synergies and potentially reduced automaticity of gait (Schlaeger et al., 2014, 2015). Further, it is likely that some of the changes that PwMS showed in biomechanical factors during gait, such as reduced propulsion and increased double support, may be attributed to conscious control of the individuals with MS to decrease risk of losing their balance. This would increase executive control of gait movements and reduce the automaticity. For the planning of tailored rehabilitation, it becomes crucial to understand how muscle activity controlled via the spinal cord in highly automatic activity such as gait is affected by alteration in motor control (Honeine et al., 2014). The identification of peculiar alterations in the pattern of activation profiles that can be tied to gait deficits is an important result of the present study.

## Limitations

This is the first study to investigate neuromotor organization in PwMS as expressed by muscle synergies. Nonetheless the study has some limitations. The extraction of muscle synergies is dependent on methodological aspects (Steele et al., 2013; Oliveira et al., 2014). Therefore, future studies should verify the consistence of the present results by improving the methodological aspect, e.g., by increasing the number of muscles and by assessing the expected similarity due to chance. Further, the number of enrolled subjects is relatively low given the heterogeneity of the subjects, even if this did not impact on the power of the statistical analysis. Finally, the results of the study can only be generalized to a population of PwMS with similar moderate mobility disabilities. A future study with a larger number of subjects could investigate if different disability severities and different subtypes of Multiple Sclerosis correlate with the amount of abnormalities in muscle synergies.

## Conclusion

Our sample of moderately affected PwMS did not differ from healthy controls in the number of muscle synergies nor in module composition walking at similar speeds. The locomotor deficits that were found to characterize gait in PwMS were instead explained by changes in activation profiles of synergies, in particular in Modules 2 and 3 that represent distal muscle activity during gait. An increase in co-activation of agonist-antagonist synergies seen during gait in PwMS compared to HS appeared not to be due to different gait velocities; conversely it was likely a strategy adopted because of compromised balance during gait.

The results of this study suggest that, analysis of muscle synergies can provide important clinical information about the functional motor deficits of people with Multiple Sclerosis and their ability to produce a desired functional outcome. Identification of inappropriate synergy activation and how this may lead to specific locomotor deficits could lead to more effective tailored rehabilitation interventions.

## Author contributions

TL, JJ, MF, DC, AC, and EG Substantial contributions to the conception or design of the work; or the acquisition, analysis, or interpretation of data for the work; and TL, JJ, and MF Drafting the work or revising it critically for important intellectual content; and TL, JJ, MF, EB, DC, AC, EG, and MR Final approval of the version to be published; and TL, JJ, MF, EB, DC, AC, EG, and MR Agreement to be accountable for all aspects of the work in ensuring that questions related to the accuracy or integrity of any part of the work are appropriately investigated and resolved.

### Conflict of interest statement

The authors declare that the research was conducted in the absence of any commercial or financial relationships that could be construed as a potential conflict of interest.

## References

[B1] Allen J. L. Kautz S. A. Neptune R. R. (2013). The influence of merged muscle excitation modules on post-stroke hemiparetic walking performance. Clin. Biomech. 28, 697–704. 10.1016/j.clinbiomech.2013.06.003 23830138 PMC3732538

[B2] Allen J. L. Neptune R. R. (2012). Three-dimensional modular control of human walking. J. Biomech. 45, 2157–2163. 10.1016/j.jbiomech.2012.05.037 22727468 PMC3405171

[B3] Benedetti M. G. Agostini V. Knaflitz M. Bonato P. (2012). Applications of EMG in Clinical and Sports Medicine, ed Steele C.(Rijeka: InTech).

[B4] Benedetti M. G. Piperno R. Simoncini L. Bonato P. Tonini A. Giannini S. (1999). Gait abnormalities in minimally impaired multiple sclerosis patients. Mult. Scler. Houndmills Basingstoke Engl. 5, 363–368. 10516781 10.1177/135245859900500510

[B5] Berg K. O. Wood-Dauphinee S. L. Williams J. I. Maki B. (1992). Measuring balance in the elderly: validation of an instrument. Can. J. Public Health Rev. Can. Sante Publique 83(Suppl. 2), S7–S11. 1468055

[B6] Berger D. J. d'Avella A. (2014). Effective force control by muscle synergies. Front. Comput. Neurosci. 8:46. 10.3389/fncom.2014.00046 24860489 PMC4029017

[B7] Bizzi E. Cheung V. C. (2013). The neural origin of muscle synergies. Front. Comput. Neurosci. 7:51. 10.3389/fncom.2013.00051 23641212 PMC3638124

[B8] Boudarham J. Hameau S. Zory R. Hardy A. Bensmail D. Roche N. (2016). Coactivation of lower limb muscles during gait in patients with multiple sclerosis. PLoS ONE 11:e0158267. 10.1371/journal.pone.0158267 27336442 PMC4919099

[B9] Bretzner F. Drew T. (2005). Contribution of the motor cortex to the structure and the timing of hindlimb locomotion in the cat: a microstimulation study. J. Neurophysiol. 94, 657–672. 10.1152/jn.01245.2004 15788518

[B10] Cameron M. H. Wagner J. M. (2011). Gait abnormalities in multiple sclerosis: pathogenesis, evaluation, and advances in treatment. Curr. Neurol. Neurosci. Rep. 11, 507–515. 10.1007/s11910-011-0214-y 21779953

[B11] Cattaneo D. Rabuffetti M. Bovi G. Mevio E. Jonsdottir J. Ferrarin M. (2014). Assessment of postural stabilization in three task oriented movements in people with multiple sclerosis. Disabil. Rehabil. 36, 2237–2243. 10.3109/09638288.2014.904933 24678992

[B12] Chalah M. A. Riachi N. Ahdab R. Créange A. Lefaucheur J.-P. Ayache S. S. (2015). Fatigue in multiple sclerosis: neural correlates and the role of non-invasive brain stimulation. Front. Cell. Neurosci. 9:460. 10.3389/fncel.2015.00460 26648845 PMC4663273

[B13] Cheron G. Duvinage M. De Saedeleer C. Castermans T. Bengoetxea A. Petieau M.. (2012). From spinal central pattern generators to cortical network: integrated BCI for walking rehabilitation. Neural Plast. 2012:375148. 10.1155/2012/375148 22272380 PMC3261492

[B14] Chervin R. D. Consens F. B. Kutluay E. (2003). Alternating leg muscle activation during sleep and arousals: a new sleep-related motor phenomenon? Mov. Disord. Off. J. Mov. Disord. Soc. 18, 551–559. 10.1002/mds.10397 12722169

[B15] Cheung V. C. Piron L. Agostini M. Silvoni S. Turolla A. Bizzi E. (2009). Stability of muscle synergies for voluntary actions after cortical stroke in humans. Proc. Natl. Acad. Sci. U.S.A. 106, 19563–19568. 10.1073/pnas.0910114106 19880747 PMC2780765

[B16] Cheung V. C. Turolla A. Agostini M. Silvoni S. Bennis C. Kasi P.. (2012). Muscle synergy patterns as physiological markers of motor cortical damage. Proc. Natl. Acad. Sci. U.S.A. 109, 14652–14656. 10.1073/pnas.1212056109 22908288 PMC3437897

[B17] Chow J. W. Yablon S. A. Stokic D. S. (2012). Coactivation of ankle muscles during stance phase of gait in patients with lower limb hypertonia after acquired brain injury. Clin. Neurophysiol. Off. J. Int. Fed. Clin. Neurophysiol. 123, 1599–1605. 10.1016/j.clinph.2012.01.006 22325644

[B18] Chvatal S. A. Ting L. H. (2013). Common muscle synergies for balance and walking. Front. Comput. Neurosci. 7:48. 10.3389/fncom.2013.00048 23653605 PMC3641709

[B19] Clark D. J. (2015). Automaticity of walking: functional significance, mechanisms, measurement and rehabilitation strategies. Front. Hum. Neurosci. 9:246. 10.3389/fnhum.2015.00246 25999838 PMC4419715

[B20] Clark D. J. Ting L. H. Zajac F. E. Neptune R. R. Kautz S. A. (2010). Merging of healthy motor modules predicts reduced locomotor performance and muscle coordination complexity post-stroke. J. Neurophysiol. 103, 844–857. 10.1152/jn.00825.2009 20007501 PMC2822696

[B21] Cofré Lizama L. E. Khan F. Lee P. V. Galea M. P. (2016). The use of laboratory gait analysis for understanding gait deterioration in people with multiple sclerosis. Mult. Scler. 10.1177/1352458516658137. [Epub ahead of print]. 27364324

[B22] d'Avella A. Giese M. Ivanenko Y. P. Schack T. Flash T. (2015). Editorial: modularity in motor control: from muscle synergies to cognitive action representation. Front. Comput. Neurosci. 9:126. 10.3389/fncom.2015.00126 26500533 PMC4598477

[B23] de Rugy A. Loeb G. E. Carroll T. J. (2013). Are muscle synergies useful for neural control? Front. Comput. Neurosci. 7:19. 10.3389/fncom.2013.00019 23519326 PMC3604633

[B24] Dominici N. Ivanenko Y. P. Cappellini G. d'Avella A. Mondi V. Cicchese M.. (2011). Locomotor primitives in newborn babies and their development. Science 334, 997–999. 10.1126/science.1210617 22096202

[B25] Fox E. J. Tester N. J. Kautz S. A. Howland D. R. Clark D. J. Garvan C.. (2013). Modular control of varied locomotor tasks in children with incomplete spinal cord injuries. J. Neurophysiol. 110, 1415–1425. 10.1152/jn.00676.2012 23761702 PMC3763159

[B26] Frank R. Larimore J. (2015). Yoga as a method of symptom management in multiple sclerosis. Front. Neurosci. 9:133. 10.3389/fnins.2015.00133 25983675 PMC4415403

[B27] Gage J. R. (1990). An overview of normal walking. Instr. Course Lect. 39, 291–303. 2186116

[B28] Givon U. Zeilig G. Achiron A. (2009). Gait analysis in multiple sclerosis: characterization of temporal-spatial parameters using GAITRite functional ambulation system. Gait Posture 29, 138–142. 10.1016/j.gaitpost.2008.07.011 18951800

[B29] Gizzi L. Nielsen J. F. Felici F. Ivanenko Y. P. Farina D. (2011). Impulses of activation but not motor modules are preserved in the locomotion of subacute stroke patients. J Neurophysiol 106, 202–210. 10.1152/jn.00727.2010 21511705

[B30] Gizzi L. Nielsen J. F. Felici F. Moreno J. C. Pons J. L. Farina D. (2012). Motor modules in robot-aided walking. J. Neuroengineering Rehabil. 9:76. 10.1186/1743-0003-9-76 23043818 PMC3533908

[B31] Gonzalez-Vargas J. Sartori M. Dosen S. Torricelli D. Pons J. L. Farina D. (2015). A predictive model of muscle excitations based on muscle modularity for a large repertoire of human locomotion conditions. Front. Comput. Neurosci. 9:114. 10.3389/fncom.2015.00114 26441624 PMC4585276

[B32] Guertin P. A. (2012). Central pattern generator for locomotion: anatomical, physiological, and pathophysiological considerations. Front. Neurol. 3:183. 10.3389/fneur.2012.00183 23403923 PMC3567435

[B33] Haines J. D. Inglese M. Casaccia P. (2011). Axonal damage in multiple sclerosis. Mt. Sinai J. Med. N. Y. 78, 231–243. 10.1002/msj.20246 21425267 PMC3142952

[B34] Hayes H. B. Chvatal S. A. French M. A. Ting L. H. Trumbower R. D. (2014). Neuromuscular constraints on muscle coordination during overground walking in persons with chronic incomplete spinal cord injury. Clin. Neurophysiol. Off. J. Int. Fed. Clin. Neurophysiol. 125, 2024–2035. 10.1016/j.clinph.2014.02.001 24618214 PMC4133333

[B35] Honeine J.-L. Schieppati M. Gagey O. Do M.-C. (2014). By counteracting gravity, triceps surae sets both kinematics and kinetics of gait. Physiol. Rep. 2:e00229. 10.1002/phy2.229 24744898 PMC3966244

[B36] Huisinga J. M. Schmid K. K. Filipi M. L. Stergiou N. (2013). Gait mechanics are different between healthy controls and patients with multiple sclerosis. J. Appl. Biomech. 29, 303–311. 10.1123/jab.29.3.303 22923390

[B37] Inman V. T. Ralston H. J. Todd F. Lieberman J. C. (1981). Human Walking. Williams & Wilkins Available online at: https://books.google.it/books?id=HNNqAAAAMAAJ

[B38] Ivanenko Y. P. Poppele R. E. Lacquaniti F. (2009). Distributed neural networks for controlling human locomotion: lessons from normal and SCI subjects. Brain Res. Bull. 78, 13–21. 10.1016/j.brainresbull.2008.03.018 19070781

[B39] Jonsdottir J. Recalcati M. Rabuffetti M. Casiraghi A. Boccardi S. Ferrarin M. (2009). Functional resources to increase gait speed in people with stroke: strategies adopted compared to healthy controls. Gait Posture 29, 355–359. 10.1016/j.gaitpost.2009.01.008 19211250

[B40] Kaipust J. P. Huisinga J. M. Filipi M. Stergiou N. (2012). Gait variability measures reveal differences between multiple sclerosis patients and healthy controls. Motor Control 16, 229–244. 10.1123/mcj.16.2.229 22615327

[B41] Kasser S. L. Jacobs J. V. Foley J. T. Cardinal B. J. Maddalozzo G. F. (2011). A prospective evaluation of balance, gait, and strength to predict falling in women with multiple sclerosis. Arch. Phys. Med. Rehabil. 92, 1840–1846. 10.1016/j.apmr.2011.06.004 21840497

[B42] Kelleher K. J. Spence W. Solomonidis S. Apatsidis D. (2010). The characterisation of gait patterns of people with multiple sclerosis. Disabil. Rehabil. 32, 1242–1250. 10.3109/09638280903464497 20156050

[B43] Kempen J. C. E. Doorenbosch C. A. M. Knol D. L. de Groot V. Beckerman H. (2016). Newly identified gait patterns in patients with multiple sclerosis may be related to push-off quality. Phys. Ther. 96, 1744–1752. 10.2522/ptj.20150508 27174257

[B44] Kesselring J. (2010). Evidence-based medicine and multiple sclerosis: figures and stories. Neuroepidemiology 35, 100. 10.1159/000310326 20551695

[B45] Kutch J. J. Valero-Cuevas F. J. (2012). Challenges and new approaches to proving the existence of muscle synergies of neural origin. PLoS Comput. Biol. 8:e1002434. 10.1371/journal.pcbi.1002434 22570602 PMC3342930

[B46] Lamontagne A. Richards C. L. Malouin F. (2000). Coactivation during gait as an adaptive behavior after stroke. J. Electromyogr. Kinesiol. Off. J. Int. Soc. Electrophysiol. Kinesiol. 10, 407–415. 10.1016/S1050-6411(00)00028-6 11102843

[B47] Lassmann H. (2013). Pathology and disease mechanisms in different stages of multiple sclerosis. J. Neurol. Sci. 333, 1–4. 10.1016/j.jns.2013.05.010 23735777

[B48] Lee D. D. Seung H. S. (1999). Learning the parts of objects by non-negative matrix factorization. Nature 401, 788–791. 10.1038/44565 10548103

[B49] Lencioni T. Jonsdottir J. Crippa A. Cattaneo D. Rovaris M. Ferrarin M. (2015). Modular organization of lower limbs in persons with multiple sclerosis and healthy persons during walking. Gait Posture 42(Suppl. 2), S14–S15. 10.1016/j.gaitpost.2015.07.035

[B50] Lizrova Preiningerova J. Novotna K. Rusz J. Sucha L. Ruzicka E. Havrdova E. (2015). Spatial and temporal characteristics of gait as outcome measures in multiple sclerosis (EDSS 0 to 6.5). J. Neuroengineering Rehabil. 12, 14. 10.1186/s12984-015-0001-0 25890382 PMC4334845

[B51] Martin C. L. Phillips B. A. Kilpatrick T. J. Butzkueven H. Tubridy N. McDonald E.. (2006). Gait and balance impairment in early multiple sclerosis in the absence of clinical disability. Mult. Scler. 12, 620–628. 10.1177/1352458506070658 17086909

[B52] Martino G. Ivanenko Y. P. d'Avella A. Serrao M. Ranavolo A. Draicchio F.. (2015). Neuromuscular adjustments of gait associated with unstable conditions. J. Neurophysiol. 114, 2867–2882. 10.1152/jn.00029.2015 26378199 PMC4737426

[B53] Michailidou I. De Vries H. E. Hol E. M. Van Strien M. E. (2015). Activation of endogenous neural stem cells for multiple sclerosis therapy. Front. Neurosci. 8:454. 10.3389/fnins.2014.00454 25653584 PMC4299409

[B54] Oliveira A. S. Gizzi L. Farina D. Kersting U. G. (2014). Motor modules of human locomotion: influence of EMG averaging, concatenation, and number of step cycles. Front. Hum. Neurosci. 8:335. 10.3389/fnhum.2014.00335 24904375 PMC4033063

[B55] Overduin S. A. d'Avella A. Carmena J. M. Bizzi E. (2012). Microstimulation activates a handful of muscle synergies. Neuron 76, 1071–1077. 10.1016/j.neuron.2012.10.018 23259944 PMC3547640

[B56] Rabuffetti M. Crenna P. (2004). A modular protocol for the analysis of movement in children. Gait Posture 20, S77–S78.

[B57] Rana M. Yani M. S. Asavasopon S. Fisher B. E. Kutch J. J. (2015). Brain connectivity associated with muscle synergies in humans. J. Neurosci. 35, 14708–14716. 10.1523/JNEUROSCI.1971-15.2015 26538643 PMC4635125

[B58] Remelius J. G. Jones S. L. House J. D. Busa M. A. Averill J. L. Sugumaran K.. (2012). Gait impairments in persons with multiple sclerosis across preferred and fixed walking speeds. Arch. Phys. Med. Rehabil. 93, 1637–1642. 10.1016/j.apmr.2012.02.019 22559932

[B59] Rodgers M. M. (1995). Dynamic foot biomechanics. J. Orthop. Sports Phys. Ther. 21, 306–316. 10.2519/jospt.1995.21.6.306 7655474

[B60] Roh J. Rymer W. Z. Beer R. F. (2015). Evidence for altered upper extremity muscle synergies in chronic stroke survivors with mild and moderate impairment. Front. Hum. Neurosci. 9:6. 10.3389/fnhum.2015.00006 25717296 PMC4324145

[B61] Roh J. Rymer W. Z. Perreault E. J. Yoo S. B. Beer R. F. (2013). Alterations in upper limb muscle synergy structure in chronic stroke survivors. J Neurophysiol 109, 768–781. 10.1152/jn.00670.2012 23155178 PMC3567389

[B62] Routson R. L. Clark D. J. Bowden M. G. Kautz S. A. Neptune R. R. (2013). The influence of locomotor rehabilitation on module quality and post-stroke hemiparetic walking performance. Gait Posture 38, 511–517. 10.1016/j.gaitpost.2013.01.020 23489952 PMC3687005

[B63] Routson R. L. Kautz S. A. Neptune R. R. (2014). Modular organization across changing task demands in healthy and poststroke gait. Physiol. Rep. 2:e12055. 10.14814/phy2.12055 24963035 PMC4208640

[B64] Rovaris M. Filippi M. Minicucci L. Iannucci G. Santuccio G. Possa F.. (2000). Cortical/subcortical disease burden and cognitive impairment in patients with multiple sclerosis. AJNR Am. J. Neuroradiol. 21, 402–408. 10696031 PMC7975362

[B65] Safavynia S. A. Ting L. H. (2013). Sensorimotor feedback based on task-relevant error robustly predicts temporal recruitment and multidirectional tuning of muscle synergies. J. Neurophysiol. 109, 31–45. 10.1152/jn.00684.2012 23100133 PMC3545166

[B66] Saltiel P. Wyler-Duda K. D'Avella A. Tresch M. C. Bizzi E. (2001). Muscle synergies encoded within the spinal cord: evidence from focal intraspinal NMDA iontophoresis in the frog. J. Neurophysiol. 85, 605–619. 11160497 10.1152/jn.2001.85.2.605

[B67] Schlaeger R. Papinutto N. Panara V. Bevan C. Lobach I. V. Bucci M.. (2014). Spinal cord gray matter atrophy correlates with multiple sclerosis disability. Ann. Neurol. 76, 568–580. 10.1002/ana.24241 25087920 PMC5316412

[B68] Schlaeger R. Papinutto N. Zhu A. H. V. Lobach I. Bevan C. J. Bucci M. (2015). Spinal Cord Gray Matter Atrophy - a Biomarker for MS Progression. Basel: ECTRIMS Online Library.

[B69] Steele K. M. Rozumalski A. Schwartz M. H. (2015). Muscle synergies and complexity of neuromuscular control during gait in cerebral palsy. Dev. Med. Child Neurol. 57, 1176–1182. 10.1111/dmcn.12826 26084733 PMC4683117

[B70] Steele K. M. Tresch M. C. Perreault E. J. (2013). The number and choice of muscles impact the results of muscle synergy analyses. Front. Comput. Neurosci. 7:105. 10.3389/fncom.2013.00105 23964232 PMC3737463

[B71] Stoquart G. Detrembleur C. Lejeune T. (2008). Effect of speed on kinematic, kinetic, electromyographic and energetic reference values during treadmill walking. Neurophysiol. Clin. Clin. Neurophysiol. 38, 105–116. 10.1016/j.neucli.2008.02.002 18423331

[B72] Tassinari C. A. Cantalupo G. Hogl B. Cortelli P. Tassi L. Francione S.. (2009). Neuroethological approach to frontolimbic epileptic seizures and parasomnias: the same central pattern generators for the same behaviours. Rev. Neurol. 165, 762–768. 10.1016/j.neurol.2009.08.002 19733874

[B73] Tassinari C. A. Rubboli G. Gardella E. Cantalupo G. Calandra-Buonaura G. Vedovello M.. (2005). Central pattern generators for a common semiology in fronto-limbic seizures and in parasomnias. A neuroethologic approach. Neurol. Sci. 26(Suppl. 3), s225–s232. 10.1007/s10072-005-0492-8 16331401

[B74] Ting L. H. Chiel H. J. Trumbower R. D. Allen J. L. McKay J. L. Hackney M. E.. (2015). Neuromechanical principles underlying movement modularity and their implications for rehabilitation. Neuron 86, 38–54. 10.1016/j.neuron.2015.02.042 25856485 PMC4392340

[B75] Ting L. H. Macpherson J. M. (2005). A limited set of muscle synergies for force control during a postural task. J. Neurophysiol. 93, 609–613. 10.1152/jn.00681.2004 15342720

[B76] Waters-Metenier S. Husain M. Wiestler T. Diedrichsen J. (2014). Bihemispheric transcranial direct current stimulation enhances effector-independent representations of motor synergy and sequence learning. J. Neurosci. 34, 1037–1050. 10.1523/JNEUROSCI.2282-13.2014 24431461 PMC3891947

[B77] Wens I. Dalgas U. Vandenabeele F. Krekels M. Grevendonk L. Eijnde B. O. (2014). Multiple sclerosis affects skeletal muscle characteristics. PLoS ONE 9:e108158. 10.1371/journal.pone.0108158 25264868 PMC4180259

[B78] Winter D. A. (1987). The Biomechanics and Motor Control of Human Gait. Waterloo, ON: University of Waterloo Press.

[B79] Winter D. A. Yack H. J. (1987). EMG profiles during normal human walking: stride-to-stride and inter-subject variability. Electroencephalogr. Clin. Neurophysiol. 67, 402–411. 2444408 10.1016/0013-4694(87)90003-4

